# Obstetric and intensive-care strategies in a high-risk pregnancy with critical respiratory failure due to COVID-19: A case report

**DOI:** 10.1016/j.crwh.2020.e00240

**Published:** 2020-07-08

**Authors:** Zuzana Kolkova, Martin F. Bjurström, John-Kalle Länsberg, Eimantas Svedas, Maria Andrada Hamer, Stefan R. Hansson, Andreas Herbst, Mehreen Zaigham

**Affiliations:** a Department of Obstetrics & Gynaecology, Institute of Clinical Sciences Lund, Lund University and Skåne University Hospital, Malmö/Lund, Sweden; b Department of Anaesthesiology and Intensive Care, Skåne University Hospital, Lund, Sweden; c Department of Clinical Sciences Lund, Paediatrics/Neonatology, Lund University, Skåne University Hospital, Malmö/Lund, Sweden

**Keywords:** SARS-CoV-2, COVID-19, Coronavirus, Pregnancy, Intubation

## Abstract

**Background:**

With the disease burden increasing daily, there is a lack of evidence regarding the impact of COVID-19 in pregnancy. Healthy pregnant women are still not regarded as a susceptible group despite physiological changes that make pregnant women more vulnerable to severe infection. However, high-risk pregnancies may be associated with severe COVID-19 disease with respiratory failure, as outlined in this report. We discuss the importance of timely delivery and antenatal steroid administration in a critically ill patient.

**Case:**

A 27-year-old pregnant woman (gravida 2, para 1) with type I diabetes, morbid obesity, hypothyroidism and a previous Caesarean section presented with critical respiratory failure secondary to COVID-19 at 32 weeks of gestation. A preterm emergency Caesarean section was performed, after steroid treatment for foetal lung maturation. The patient benefited from prone positioning; however, transient acute renal injury, rhabdomyolysis and sepsis led to prolonged intensive care and mechanical ventilation for 30 days. The baby had an uncomplicated recovery.

**Conclusion:**

COVID-19 infection in high-risk pregnancies may result in severe maternal and neonatal outcomes such as critical respiratory failure requiring mechanical ventilation and premature termination of the pregnancy. Antenatal steroids may be of benefit for foetal lung maturation but should not delay delivery in severe cases.

## Highlights

• Severe COVID-19 in high-risk pregnancies can lead to acute respiratory failure requiring mechanical ventilation and preterm delivery
• A multidisciplinary team may be vital in reducing adverse maternal and neonatal outcomes
• Antenatal steroids may be of benefit for foetal lung maturation but should not delay delivery in severe cases.
• Acute renal failure, rhabdomyolysis and sepsis can complicate recovery from COVID-19

## 1 Introduction

The SARS-CoV-2 virus has produced an unprecedented global health crisis. There are particularly vulnerable groups within society. In pregnant women the case morbidity rate is up to 3% [1] and the mortality rate is 1.2% [2]. Although pregnant women are more prone to viral infections, health agencies have been seemingly reluctant to identify them as a susceptible group [3,4]. We present a case of severe COVID-19 in pregnancy leading to preterm Caesarean delivery and critical respiratory failure with intensive-care treatment of both mother and newborn.

## 2 Case

A 27-year-old woman (gravida 2, para 1) was transferred from her local county hospital to the regional university hospital at gestational week (GW) 32 + 1 due to a positive throat swab for SARS-CoV-2 (quantitative real-time polymerase chain reaction), increasing oxygen demand and a lack of intensive-care beds for COVID-19.

**Fig. 1:**
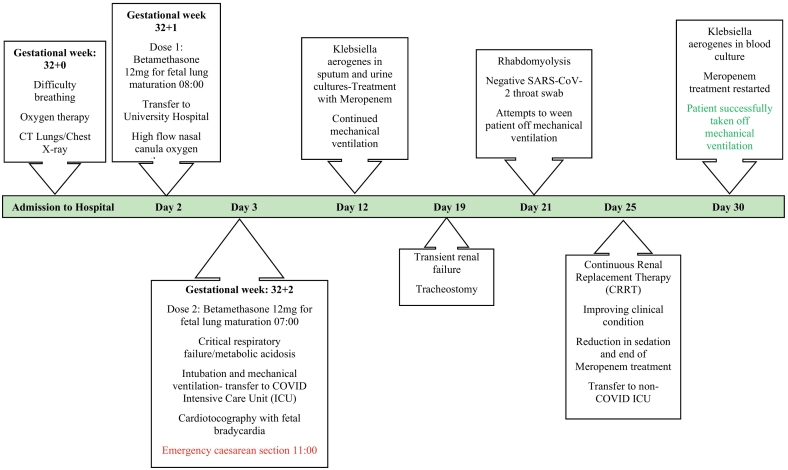
Timeline of course of disease in patient.

**Fig. 2:**
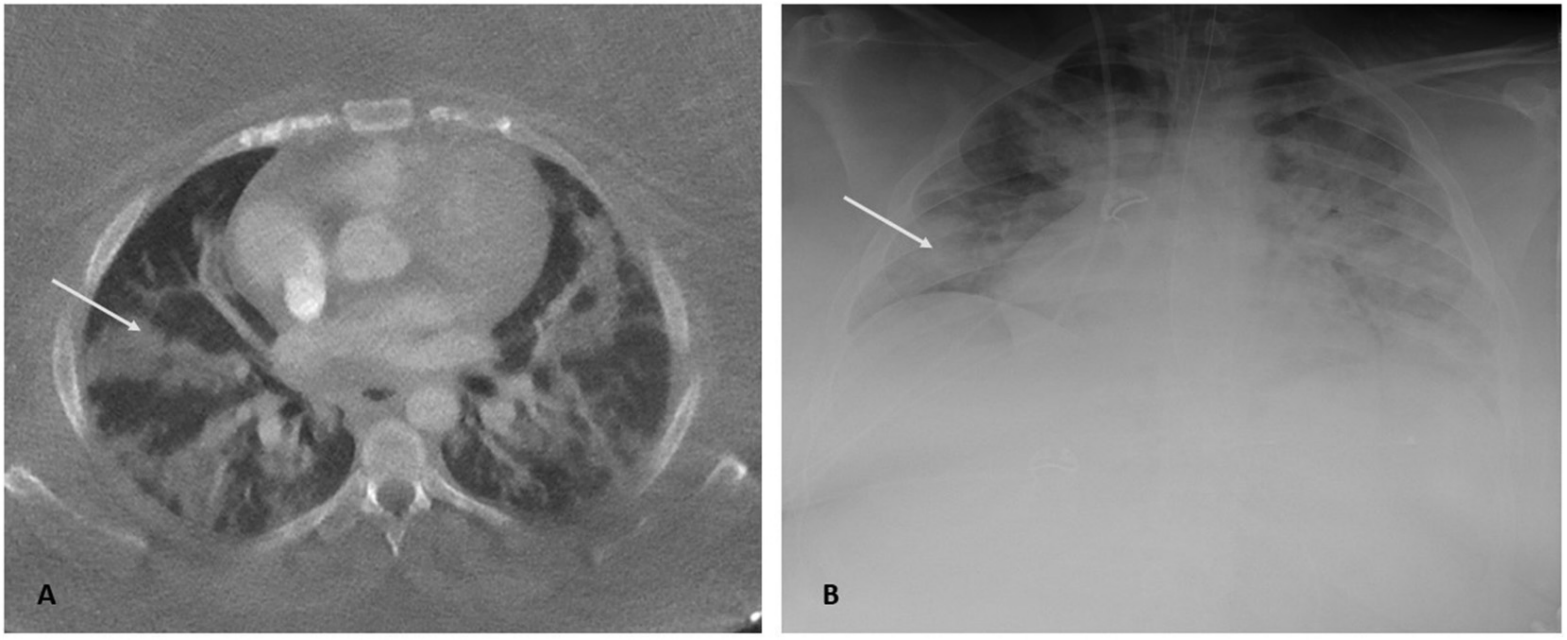
**A.** Low-dose computed tomography axial scan showing bilateral multifocal ground-glass opacities, with both peripheral and perihilar distribution, corresponding to COVID-19 pneumonia. **B.** Chest radiograph showing bilateral opacities and signs of congestion.

The patient, a pre-school teacher of Middle Eastern descent, had a seven-day history of fever, lower abdominal pain, malaise, headache, cough, dyspnoea and polyuria (Fig. 1). The patient suffered from extreme obesity (BMI 57 kg/m^2^), poorly regulated type-1 diabetes mellitus and hypothyroidism. She had been prescribed acetylsalicylic acid (160 mg daily) due to preeclampsia in her previous pregnancy; delivery had been via Caesarean section at GW 36. During the current pregnancy, an obstetric ultrasound showed a foetal weight deviation of +32% (LGA: large for gestational age) at GW 29 + 6. Thromboprophylaxis (8000 IE tinzaparin), with an initial dose of betamethasone (12 mg intra-muscular) for foetal lung maturation, was given at the local hospital. A chest computed tomography (CT) scan revealed bilateral diffuse ground-glass opacities with no signs of pulmonary embolism (Fig. 2).

At the university hospital, a multidisciplinary team of obstetricians, anaesthesiologists and neonatologists started to prepare for Caesarean delivery. The tinzaparin dose was doubled to 16,000 IE divided in two doses daily and a normal cardiotocograph (CTG) was registered. The respiratory condition of the patient deteriorated during the night and despite oxygen at 100% (high flow nasal cannula, HFNC) and gas flow at 60–80 L/min, the oxygen saturation fell below 90% and critical respiratory failure with metabolic acidosis ensued (Fig. 1). The patient was subsequently intubated and put on mechanical ventilation at the COVID intensive-care unit (ICU). The second 12 mg dose of betamethasone for foetal lung maturation was administered, with the plan to perform an emergency Caesarean section. After stabilisation, the CTG showed reduced variability and recurring episodes of foetal bradycardia associated with the patient's positioning. An emergency Caesarean was performed at GW 32 + 2, 8 days after the onset of the respiratory symptoms and 4 h after intubation. The operation was technically challenging due to extreme obesity and intraabdominal adhesions. Piperacillin/tazobactam was administered preoperatively and the total blood loss was 200 ml.

Oxygenation was critically impaired during the first two days in the ICU. During days 1–9 of the intensive-care period, the patient required prone positioning and intermittent muscle relaxation to optimise respiration and to provide lung-protective ventilation (Table 2). To treat muco-purulent secretions interfering with ventilation, aerosolized dornase-alfa was used. No antiviral treatment was administered. In addition to the respiratory failure, the patient developed acute renal injury. Due to persistent high fever, continuous renal replacement therapy was used for invasive cooling in order to maintain adequate temperature control. A nosocomial superinfection with *Klebsiella aerogenes* was detected in tracheal secretions, urine cultures and later on in blood (Fig. 1). Treatment with meropenem (1 g × 3 daily) was initiated. Due to a prolonged ICU course and palpable stress, cough, high fever, and a lack of contact during wake-up tests, a tracheostomy was performed on day 19, to facilitate weaning from mechanical ventilation. Rhabdomyolysis ensued on days 21–23, which further complicated the recovery period (Table 1). Tracheal swabs for SARS-CoV-2 returned repeatedly negative and the patient was transferred to the non-COVID ICU. The patient was successfully taken off mechanical ventilation 30 days after her first day of admission.

**Table 1:** Maternal laboratory values during intensive care.

Variable	Normal Reference Range	Day 3 (Emergency Caesarean Section)	Day 4 (Day after Caesarean Section)	Day 23 of Intensive Care
Haemoglobin (Hb) g/L	117–153	93	86	80
Platelet count x10^9^/L	165–387	190	152	314
White cell count x10^9^/L	3.5–8.8	10.1	9.0	10.1
Neutrophil count x10^9^/L	1.8–7.5	9.0	7.8	7.4
Lymphocyte count x10^9^/L	1.0–4.0	0.5	0.2	2.0
Ferritin μmol/L	13–148	666	366	254
C-reactive protein (CRP) mg/L	<5	157	222	29
Procalcitonin μg/L	< 0.05	1.3	5.2	1.4
Troponin-T ng/L	<5	5	7	141
Myoglobin μg/L	25–58	26	–	13,732
Glucose mmol/L	4.2–6.0	7.9	10.7	5.8
Aspartate aminotransferase (ASAT) μkat/L	0.25–0.6	16	12	2.8
Alanine aminotransferase (ALAT) μkat/L	0.15–0.75	3.6	3.5	4.4
Alkaline phosphatase (ALP) μkat/L	0.70–1.9	1.9	1.3	1.3
Gamma-glutamyl transferase (GGT) μkat/L	0.15–0.75	1.5	1.2	3.8
Bilirubin μmol/L	5–25	9	15	6
Lactate Dehydrogenase (LDH) μkat/L	1.8–3.4	19	14	11
Pancreatic amylase μkat/L	0.15–1.1	0.67	0.79	0.35
Plasma Albumin g/L	36–48	21	–	25
Estimated Glomerular Filtration Rate (eGFR)	80–125	38	24	14
Creatinine μmol/L	45–90	116	119	338
Urea mmol/L	2.6–6-4	5.1	6.4	39.4
Sodium mmol/L	137–145	141	143	145
Potassium mmol/L	3.5–4.4	4.8	4.1	5.0
Chloride mmol/L	98–110	114	111	98
Calcium ion mmol/L	1.15–1.33	1.20	1.21	1.27
Magnesium mmol/L	0.70–0.95	0.69	0.94	0.94
Prothrombin-complex International Normalized Ratio (P-INR)	0.9–1.2	1.0	1.0	0.9
Activated Partial Thromboplastin Time (APTT) in seconds (s)	26–33	45	40	31
D-Dimer				2.6
Fibrinogen g/L	2.0–4.0	5.4	5.8	6.0
pH	7.35–7.45	7.18	7.36	7.46
Partial pressure of carbon dioxide pCO_2_ in kPa	4.6–6.0	5.4	5.9	6.2
Partial pressure of oxygen pO_2_ in kPa	10.0–13.0	6.7	9.3	9.1
Base Excess mmol/L	22–27	14	23	32
Bicarbonate HCO_3_^−^ mmol/L	−3.0-3.0	−12.2	−0.9	+8.4
Lactate mmol/L	0.5–1.6	2.9	3.8	1.9
Saturation of oxygen %	97–100	77	93	93

**Table 2:** Mechanical ventilation respiratory parameters during the first two weeks of intensive care.

	Day 1	Day 2	Day 3	Day 4	Day 5	Day 6	Day 7	Day 8	Day 9	Day 10	Day 11	Day 12	Day 13	Day 14
Prone Ventilation	Yes	Yes	Yes	Yes	Yes	Yes	Yes	Yes	Yes	No	No	No	No	No
Muscle relaxation	Yes	Yes	Yes	Yes	Yes	Yes	Yes	Yes	No	No	No	No	No	No
FiO_2_	0.65–0.1	0.5–0.8	0.45–0.8	0.5–0.8	0.5–0.7	0.4–0.8	0.35–0.6	0.35–0.45	0.45–0.55	0.35–0.5	0.3–0.5	0.3–0.45	0.4–0.45	0.4–0.6
PEEP	14–15	14–15	12–15	12–14	14–16	14–16	16	11–16	10–14	11–12	10–12	8–10	8–10	8–10
PF-ratio	8–15	13–20	15–21	10–20	12–22	16–25	22–31	19–32	15–24	19–27	23–27	21–26	19–24	20–26

Abbreviations: D = day, FiO_2_ = fraction of inspired oxygen, PEEP = positive end-expiratory pressure, P/F = arterial oxygen partial pressure (kPa) / FiO2.

P/F ratio: ≤39.9 = mild acute respiratory distress syndrome (ARDS), ≤26.6 = moderate ARDS, ≤13.3 = severe ARDS.

**Table 3:** Neonatal Apgar Score at 1, 5 and 10 min.

	1 min	5 min	10 min
Activity	0	0	1
Pulse	2	2	2
Grimace	0	1	1
Appearance	1	1	2
Respiration	0	1	2
Total Apgar score	3	5	8

The neonate, a boy weighing 3100 g (99th percentile), had absent tone and lack of spontaneous breathing (Table 3). Manual ventilation was initiated, after which the heart rate and oxygen saturation stabilised promptly. After 6 min, spontaneous breathing was established. Nasal continuous positive airway pressure (nCPAP) with positive end expiratory pressure (PEEP) at 5 cmH_2_O and 30% oxygen was applied. Upon arrival at the neonatal ICU, umbilical artery and vein catheters were inserted. Arterial cord blood gas analysis showed mild respiratory acidosis (pH 7.21, pCO_2_ 8.9 kPa) at birth. During the catheterisation procedure, the need for oxygen increased from fraction of inspired oxygen (FiO_2_) 0.3 to 0.6, presenting with deep intercostal retractions. A chest X-ray showed atelectasis of the inferior right lung lobe. Nasal intubation was performed, and volume-targeted conventional mechanical ventilation was initiated. Surfactant (Poractant alfa®) 200 mg/kg was given intratracheally. The FiO_2_ decreased incrementally over the following 12 h and the neonate was extubated after 24 h. No further breathing support was needed. Nasal swabs for SARS-CoV-2 were negative at 48 and 96 h postpartum.

## 3 Discussion

We report a critical case of COVID-19 in a high-risk pregnancy, with acute respiratory failure requiring mechanical ventilation and premature termination of the pregnancy. Although pregnant women are not recognised as a vulnerable group for COVID-19, there is a growing body of evidence linking late pregnancy and prior maternal risk factors such as high BMI, diabetes and hypertension to adverse pregnancy outcomes, including maternal and neonatal deaths [1,2,[5], [6], [7]].

The patient presented with several risk factors that have been linked to an increased likelihood of a severe course for COVID-19, including morbid obesity (BMI 57 kg/m^2^), diabetes mellitus [7] and Asian origin [8]. Ethnicity has been implicated due to a general higher prevalence of medical problems such as cardiovascular disease, diabetes and higher deprivation in such groups. In a cohort of hospitalised cases in the United States, peak respiratory support for severe COVID-19 in pregnancy has been reported to occur on day 8 and intubation on day 9 [7]. Co-morbidities like previous pulmonary/cardiac disease and high BMI were again associated with severe disease.

A multidisciplinary team opted to complete antenatal steroid therapy for foetal lung maturation since the foetus was at risk for respiratory distress (GW 32, LGA and poorly regulated maternal diabetes). Some reports have warned against the use of corticosteroids in critically ill patients, due to risk of delivery postponement and worsening of the clinical course [9], including delayed viral clearance. The International Society of Ultrasound in Obstetrics and Gynaecology advises against antenatal steroid treatment in preterm COVID-19 cases (GW 34–36) and recommends caution at earlier stages of gestation [10]. In contrast, the Swedish Federation of Obstetricians and Gynaecologists supports the use of antenatal steroids before GW 34 in COVID-19 cases [11]. The RECOVERY trial [12] reported a reduction in ICU deaths by one-third in ventilated COVID-19 patients receiving dexamethasone therapy. These findings were supported by another recent study where early administration of dexamethasone was found to reduce the duration of mechanical ventilation and overall mortality in patients with moderate to severe respiratory failure [13]. Further investigation of the potential risks and benefits of antenatal steroid treatment in severe COVID-19 cases in pregnancy is therefore warranted.

The patient was mechanically ventilated for about 4 h prior to the Caesarean section and put in prone position 2 h after surgery. Swedish guidelines recommend delivery within 24 h in cases where the mother requires more than 5 l oxygen [11]. In this case, a multidisciplinary team decided to postpone delivery in order to temporarily stabilise the respiratory condition of the patient and complete steroid treatment for foetal lung maturity. It remains unclear whether an earlier Caesarean section could have prevented the patient from critical respiratory failure.

Serum interleukin-6 (IL-6) levels peaked on day 1 of the ICU period (2378 ng/L) and remained below 90 ng/L from day 3 onwards. Levels of other acute phase proteins (APPs) such as fibrinogen, ferritin and C-reactive protein were also elevated, although no clear dynamics were seen during the first 19 days of intensive care. Hyperactive immune responses characteristic of severe COVID-19 have been shown to cause stress-induced tissue injury and multi-organ impairment [14]. Elevated levels of IL-6 have been associated with an increased risk of mortality [15]. The APP and liver enzyme levels improved drastically after delivery, suggesting that severe COVID-19 infection during pregnancy may improve after delivery [16].

Prior poor health, nosocomial infection followed by acute renal failure, rhabdomyolysis and sepsis led to prolonged ICU care. Rhabdomyolysis has been presented as a possible late complication of COVID-19 although other infections, drug interactions, hypoxemia, extremes of body temperature etc. may also have been implicated [17]. To the best of our knowledge, this is the first report of COVID-19 with subsequent rhabdomyolysis postpartum.

In summary, this case report describes the obstetric and intensive-care management of a critical case of COVID-19 in the third trimester. We discuss the timing of delivery and the role of antenatal steroid treatment for foetal lung maturation, which may be factors important for future recommendations regarding severe COVID-19 in pregnancy.
